# Combination of Radiation Therapy, Wilms' Tumor 1 (WT1) Dendritic Cell Vaccine Therapy, and α-Galactosylceramide-Pulsed Dendritic Cell Vaccine Therapy for End-Stage Small Bowel Cancer

**DOI:** 10.7759/cureus.64972

**Published:** 2024-07-20

**Authors:** Hisashi Nagai, Hao Chen, Ryusuke Karube, Yusuke Koitabashi, Ouka Numata, Kenichi Yamahara

**Affiliations:** 1 Department of Human Development - Environment and Resources, Graduate School of Human and Environmental Studies, Tokai University, Kanagawa, JPN; 2 Department of Regenerative Medicine, Ginza Phoenix Clinic, Tokyo, JPN; 3 Department of Respiratory Medicine, Yokohama City University Hospital, Yokohama, JPN; 4 Laboratory of Molecular and Cellular Therapy, Institute for Advanced Medical Sciences, Hyogo Medical University, Hyogo, JPN

**Keywords:** cancer immunotherapy, end-stage cancer, immune cell therapy, oncology, small intestine cancer

## Abstract

There is no established treatment for terminal cancer patients who no longer respond to surgery, radiotherapy, or chemotherapy, and palliative care is the standard worldwide. We performed intensity-modulated radiation therapy for pain relief in a 40-year-old male patient with end-stage small intestinal cancer who had been diagnosed with a life expectancy of two months after chemotherapy had been ineffective. Subsequent administration of seven doses of dendritic cell vaccine recognizing Wilms' tumor 1 (WT1) and α-galactosylceramide antigens resulted in significant shrinkage of the cancer and marked improvement of the patient's general condition. The combination therapy of radiotherapy and dendritic cell vaccine therapy may suppress cancer progression and prolong survival, even in patients with chemotherapy-refractory terminal cancer. In particular, double dendritic cell vaccine therapy with WT1 and α-galactosylceramide-pulsed dendritic cell may provide an anti-tumor immune effect that is superior to that of the respective monotherapy.

## Introduction

Surgery and radiotherapy are not indicated for stage IV small intestinal cancer, and FOLFOX (folinic acid, fluorouracil, oxaliplatin) therapy is the first choice. When FOFOX is not effective, chemotherapy, such as nab-paclitaxel and panitumumab, is used, but their efficacy is limited [1]. If chemotherapy is not effective, the patient is generally transferred to palliative care medicine.

There are three standard treatments for cancer: surgery, radiation therapy, and chemotherapy.

In addition to these, immunotherapy has attracted attention as the fourth standard therapy, and immune checkpoint inhibitors against PD-1, PD-L1, and CTLA-4 are now being implemented worldwide. On the other hand, therapies using the patient's own immune cells for solid tumors are still under development [2].

Dendritic cell vaccine therapy is a type of immune cell therapy in which specific cancer antigens are presented to T cells to increase anti-tumor immunity in the patient's body. Among the many cancer antigens such as MUC1, Her-2, MAGE3, and p53, Wilms’ tumor 1 (WT1) has been shown to be highly expressed in a large number of cancer types, and the National Cancer Institute has listed WT1 as the most clinically effective common cancer antigen [3]. We have used WT1-dendritic cell vaccine (WT1-DC), in which WT1 is recognized by dendritic cells ex vivo, for treatment and have reported successful responses in late-stage cancers that have failed chemotherapy [4-7].

On the other hand, dendritic cells that recognize the glycolipid antigen α-galactosylceramide (α-Galcer) have been reported to activate natural killer T (NKT) cells in patients, and directly or indirectly NKT cells enhance antitumor immunity, and several clinical trials have been already conducted [8]. However, there are no reports on the combination therapy of WT1-DC and α-Galcer-DC.

In addition, the concept of immunoradiotherapy, which combines radiotherapy and immunotherapy, has recently attracted attention, with some reports suggesting that the combination of irradiation and immune checkpoint inhibitors is useful [9,10]. However, there are very few reports combining radiation therapy with immunotherapy, and none for small bowel cancer.

We report a case in which radiotherapy, WT1-DC, and α-Galcer-DC were effective as a combination in a terminal small intestinal cancer with a life expectancy of two months, in which chemotherapy was ineffective.

## Case presentation

The patient is a 40-year-old male with a past medical history of diagnosis of dermatomyositis due to skin rash and myalgia on his extremities. He was positive for anti-TIFγ antibody, and after examination for malignancy, he was diagnosed with small intestinal cancer with para-aortic lymph node metastasis and peritoneal dissemination. He was treated with first-line FOLFOX + bevacizumab (Bev), second-line encorafenib + cetuximab, third-line FOLFIRI (folic acid, fluorouracil, irinotecan) + Bev, and fourth-line trifluridine/tipiracil (FTD/TPI) + Bev as chemotherapy, but due to cancer progression, the patient developed duodenal and bile duct obstruction and was treated with bile duct stenting and central venous nutrition.

The patient had severe abdominal cancer pain and underwent single palliative intensity-modulated radiation therapy (IMRT) at 8 Gy, but still pain control was poor. At this point, he was diagnosed with a life expectancy of two months. He was wheelchair-bound and had a performance status (PS) of 4. He continued chemotherapy with FTD/TPI + Bev.

The patient presented to our clinic in February 2024. Six sessions of WT1-DC and α-Galcer-DC + nivolumab 20 mg infusion therapy were given at our clinic starting three weeks after IMRT irradiation, with two sessions of activated lymphocyte therapy in between. The duration of treatment was approximately three months.

WT1-DC was administered subcutaneously in the right and left paraganginal lymph nodes, and α-Galcer-DC was administered intravenously. The maximum diameter of delayed type hypersensitivity (DTH) to the antigen was measured as DTH max using the conventional method.

**Figure 1:**
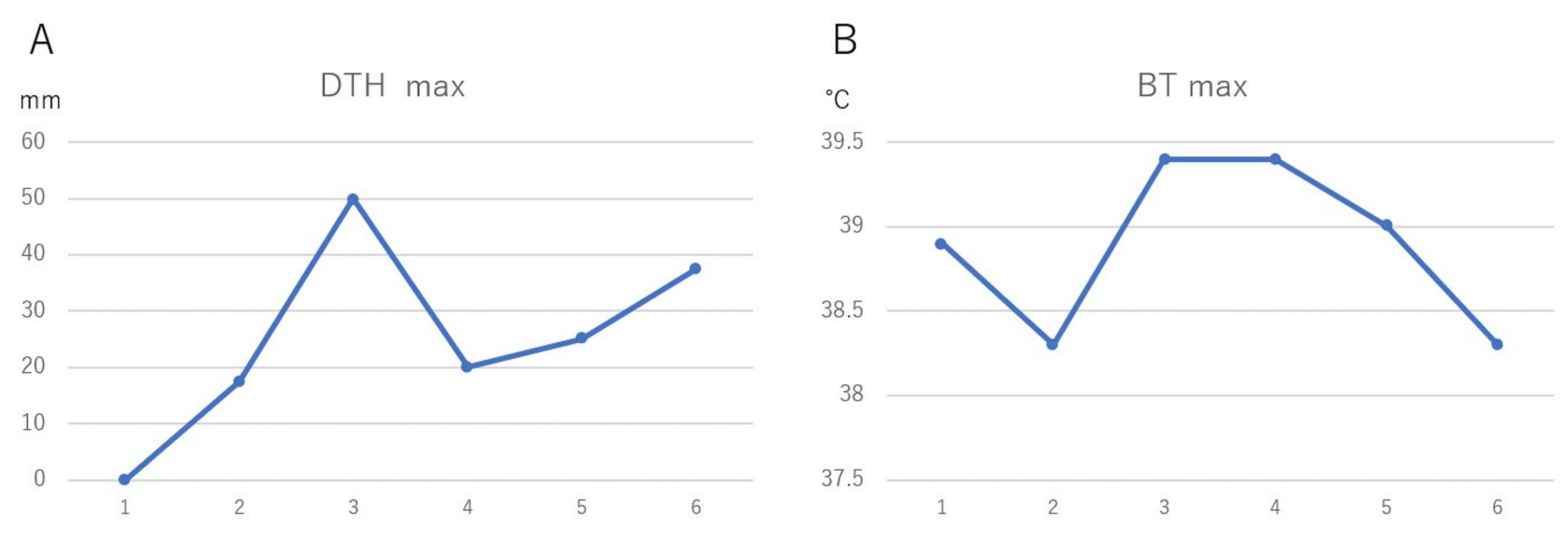
Change in immune response to WT1 antigen DTH and temperature max within three days after simultaneous administration of WT1-DC and α-Galcer-DC. The x-axis represents the number of dendritic cells administered. DTH max was less than 40 mm in diameter except for the third dose. BT showed a high fever of 38℃ or higher at all times. DTH, delayed type hypersensitivity; BT, body temperature; WT1, Wilms' tumor 1; WT1-DC, Wilms' tumor 1-dendritic cell vaccine; α-Galcer-DC, α-galactosylceramide-dendritic cell vaccine

Although a high fever with a maximum body temperature of 38.9℃ was observed within three days after the first dose of DC vaccine, DTH was not observed. The patient consistently had a high fever of 38℃ or higher until the sixth dose of DC vaccine. On the other hand, DTH max could be measured after the second dose, but was less than 40 mm in diameter except for the third dose (Figure 1).

**Figure 2:**
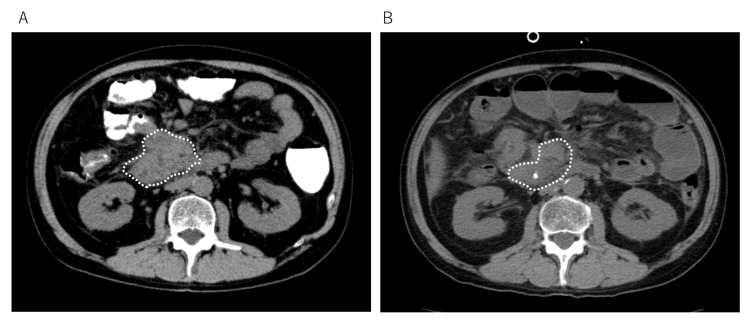
Change in cancer size before and after immunotherapy Abdominal CT findings before (A) and after (B) immunotherapy with WT1-DC and α-Galcer-DC. The dotted line indicates the small intestine cancer, which shrank significantly after three months of treatment. WT1-DC, Wilms' tumor 1-dendritic cell vaccine; α-Galcer-DC, α-galactosylceramide-dendritic cell vaccine

Abdominal CT scan before and after three months of treatment showed significant reduction of small intestinal cancer (Figure 2). In addition, blood tests showed a marked decrease in CA19-9, a decrease in bilirubin, and improvement in liver function (Table 1). With these changes, the patient's abdominal pain decreased. He was able to resume a liquid diet, and his PS improved to 2. Currently, he walks to the hospital on his own without a cane and is able to perform basic daily activities such as toileting and bathing on his own.

**Table 1:** Change in blood test data before and after immunotherapy Comparison of blood test data before and after immunotherapy with WT1-DC and α-Galcer-DC. Liver function improved with shrinkage of the small intestinal cancer, and there was a marked decrease in CEA and CA19-9. Neutrophil percentage was mildly decreased and lymphocyte percentage was mildly increased. WBC, white blood cell; RBC, red blood cell; Hb, hemoglobin; Plt, platelet; Nue, neutrophil ratio; Lym, lymphocyte ratio; Alb, albumin; T-Bil, total bilirubin; D-Bil, direct bilirubin; AST, aspartate aminotransferase; ALT, alanine aminotransferase; γGTP, γ-glutamyl transpeptidase; Cr, creatinine; CRP, C-reactive protein; CEA, carcinoembryonic antigen; CA19-9, carbohydrate antigen 19-9; CA72-4, cancer antigen 72-4; WT1-DC, Wilms' tumor 1-dendritic cell vaccine; α-Galcer-DC, α-galactosylceramide-dendritic cell

Data item	Before treatment	After treatment	Unit	Reference range
WBC	11,130	6,300	/uL	3,500-9,700
RBC	432	378	×10^4^/uL	438-577
Hb	13.1	11.9	g/dL	13.6-18.3
Plt	32.6	26.6	×10^4^/uL	14.0-37.9
Neu	91	88	%	42.0-74.0
Lym	3	6	%	18.0-50.0
Alb	3.7	3.8	g/dL	3.8-5.2
T-Bil	0.9	0.4	mg/dL	0.3-1.2
D-Bil	0.6	0.2	mg/dL	<0.4
AST	64	37	U/L	10-40
ALT	136	46	U/L	5-45
γGTP	398	129	U/L	<79
Cr	0.46	0.57	mg/dL	0.65-1.09
CRP	0.17	0.12	mg/dL	<0.30
Ferritin	607.8	446.3	ng/mL	21-282
CEA	3.7	2.7	ng/mL	<5.0
CA19-9	3122.5	75	U/mL	<37.0
CA72-4	0.8	0.8	U/mL	<6.9

## Discussion

The patient had an extremely poor prognosis with cancer of the small intestine that had not responded to chemotherapy, had reached the fourth line, and had bile duct and duodenal obstruction. The patient's blood count prior to the start of immunotherapy was more than 10,000 in white blood cells, 91% neutrophils, and 3% lymphocytes. These show an extremely unbalanced immune cell population. His blood CRP and ferritin levels were high, and his CA19-9 level was 3122.5 U/mL (reference range <75U/mL), indicating a strong inflammatory response and active cancer growth.

Patients with WT1-specific cytotoxic T cells induced in DC vaccine targeting the WT1 antigen often show DTH max greater than 40 mm after repeated WT1-DC administration [5]. However, the first dose in this case showed 0 mm, and except for the third dose, the diameter was less than 40 mm, indicating a weak immune response. On the other hand, the febrile response was extremely good, with temperatures above 38℃ in all six doses, which is also a high level for a successful DC vaccine case. One possible reason for the marked shrinkage of the cancer, despite the discrepancy between DTH and temperature trends, is that α-Galcer-DC was used in combination with WT1-DC in this case.

α-Galactosylceramide is a glycolipid, and dendritic cells that recognize it have been reported to activate NKT cells in the body, leading to induce not only direct cancer cell damage by NKT cells but also gross anti-tumor immunity, including activating CD8+ T cells and NK cells [8,11]. In cancer patients, NKT cell function is impaired and their numbers are low, making isolation and culture technically difficult. Therefore, indirect NKT cell activation therapy by administration of α-Galcer-DC is often performed as "NKT cell therapy” [12]. Since there is no common evaluation method of immune activation by α-Galcer-DC vaccine in clinical practice, it is not possible to determine the exact impact of this method. In our experience, when a clinical response is observed with WT1-DC, both DTH max and body temperature (BT) max are always high, suggesting that α-Galcer-DC may have compensated for the deficiency of WT1-DC in the present case. Therefore, the combination therapy of WT1-DC and α-Galcer-DC may be useful even in highly malignant cancers. To evaluate this, it is necessary to develop a clinical evaluation index for α-Galcer-DC.

One noteworthy aspect of this case is that immunotherapy was initiated three weeks after the implementation of 8 Gy single IMRT for palliative purpose for refractory abdominal pain. Although 8 Gy single IMRT is currently the standard method of radiotherapy for pain palliation, when low-dose irradiation is used in combination with cancer immunotherapy, the in vivo cancer vaccination effect by activation of cancer immunogenicity is attracting attention. This is a method called immunoradiation therapy, in which irradiation is not aimed at cancer necrosis but rather to elevate anti-tumor immune response through damage to cancer cell genes and the generation of cancer-specific antigens in the repair process and exposure of cancer antigens stored inside cancer cells by increased expression of MHC (major histocompatibility complex) class 1 on the cell membrane [13,14].

In this case, a single 8 Gy IMRT was coincidentally performed three weeks before the immunotherapy, which may have matched the timing of the increase in cancer immunogenicity and the activation of the immune cells. We have already reported such a case in which a large cervical cancer liver metastasis was significantly reduced by immunotherapy administered one month after IMRT [7]. Since it is theoretically possible that cancer immunogenicity may decrease again when cancer cells fully recover from irradiation-induced cancer damage, a detailed study on the timing of the combination of irradiation and immunotherapy is necessary.

We report a case in which IMRT and two types of dendritic cell vaccine therapies for terminal small bowel cancer resulted in tumor shrinkage and a marked improvement in overall health. This case suggests the usefulness of immunoradiotherapy. On the other hand, since conclusions cannot be drawn from a single case alone, validation in a large-scale clinical trial with a larger number of patients is needed.

## Conclusions

We report a case in which a combination of WT1-DC and α-Galcer-DC was effective in shrinking cancer in a patient with small intestinal cancer who had been diagnosed with a life expectancy of three months after failure of chemotherapy. WT1-DC alone may not have been effective enough, but α-Galcer-DC may have complemented it. In addition, the combination may have provided a very clinically meaningful result, as cancer immunogenicity was increased by the coincidental administration of a single 8 Gy IMRT three weeks prior to the start of immunotherapy. The combination of multiple immunotherapy and radiotherapy has the potential to push the limits of standard treatment of cancer.

## References

[1] Overman MJ (2009). Recent advances in the management of adenocarcinoma of the small intestine. Gastrointest Cancer Res.

[2] Zhang Y, Zhang Z (2020). The history and advances in cancer immunotherapy: understanding the characteristics of tumor-infiltrating immune cells and their therapeutic implications. Cell Mol Immunol.

[3] Cheever MA, Allison JP, Ferris AS (2009). The prioritization of cancer antigens: a national cancer institute pilot project for the acceleration of translational research. Clin Cancer Res.

[4] Nagai H, Karube R, Zhao F (2023). Radioimmunotherapy with WT1 dendritic cell vaccine for end-stage lung adenocarcinoma markedly shrinks tumors. Cureus.

[5] Nagai H, Karube R (2023). Delayed-type hypersensitivity: an excellent indicator of anti-tumor immunity with Wilms' tumor 1 (WT1) dendritic cell vaccine therapy. Cureus.

[6] Nagai H, Karube R (2023). WT1 dendritic cell vaccine therapy improves immune profile and prolongs progression-free survival in end-stage lung cancer. Cureus.

[7] Nagai H (2024). Immunoradiation therapy for end-stage undifferentiated cervical cancer that restored sensitivity to chemotherapy and resulted in the disappearance of the cancer. Cureus.

[8] Wolf BJ, Choi JE, Exley MA (2018). Novel approaches to exploiting invariant NKT cells in cancer immunotherapy. Front Immunol.

[9] Mondini M, Levy A, Meziani L, Milliat F, Deutsch E (2020). Radiotherapy-immunotherapy combinations - perspectives and challenges. Mol Oncol.

[10] Shevtsov M, Sato H, Multhoff G, Shibata A (2019). Novel approaches to improve the efficacy of immuno-radiotherapy. Front Oncol.

[11] Zhang Y, Springfield R, Chen S (2019). α-GalCer and iNKT cell-based cancer immunotherapy: realizing the therapeutic potentials. Front Immunol.

[12] Nelson A, Lukacs JD, Johnston B (2021). The current landscape of NKT cell immunotherapy and the hills ahead. Cancers (Basel).

[13] Menon H, Chen D, Ramapriyan R (2019). Influence of low-dose radiation on abscopal responses in patients receiving high-dose radiation and immunotherapy. J Immunother Cancer.

[14] Demaria S, Guha C, Schoenfeld J (2021). Radiation dose and fraction in immunotherapy: one-size regimen does not fit all settings, so how does one choose?. J Immunother Cancer.

