# Correction: Combination of Radiation Therapy, Wilms' Tumor 1 (WT1) Dendritic Cell Vaccine Therapy, and α-Galactosylceramide-Pulsed Dendritic Cell Vaccine Therapy for End-Stage Small Bowel Cancer

**DOI:** 10.7759/cureus.c220

**Published:** 2025-05-05

**Authors:** Hisashi Nagai, Hao Chen, Ryusuke Karube, Yusuke Koitabashi, Ouka Numata, Kenichi Yamahara

**Affiliations:** 1 Department of Human Development - Environment and Resources, Graduate School of Human and Environmental Studies, Tokai University, Kanagawa, JPN; 2 Department of Regenerative Medicine, Ginza Phoenix Clinic, Tokyo, JPN; 3 Department of Respiratory Medicine, Yokohama City University Hospital, Yokohama, JPN; 4 Laboratory of Molecular and Cellular Therapy, Institute for Advanced Medical Sciences, Hyogo Medical University, Hyogo, JPN

This article has been corrected as follows. "Wilms' tumor" was originally and erroneously written as "Wim's tumor" throughout the article. This has now been corrected. The journal editorial staff regrets that this error was not identified prior to publication.

